# Supplementary material for: Multi‐dataset identification of innovative feature genes and molecular mechanisms in keratoconus
Source: J Cell Mol Med. 2024 Sep 19;28(18):e70079. doi: 10.1111/jcmm.70079 (PMC11412914; doi:10.1111/jcmm.70079)
Supplement: Supplementary file 2 — Material S2. [file JCMM-28-e70079-s002.zip › 7.GSEA analysis/1.ARL11/KEGG_ARRHYTHMOGENIC_RIGHT_VENTRICULAR_CARDIOMYOPATHY_ARVC.html]

Details for gene set KEGG\_ARRHYTHMOGENIC\_RIGHT\_VENTRICULAR\_CARDIOMYOPATHY\_ARVC[GSEA]

|  || Dataset | ARL11.group.cls#h\_versus\_l |
| Phenotype | group.cls#h\_versus\_l |
| Upregulated in class | l |
| GeneSet | KEGG\_ARRHYTHMOGENIC\_RIGHT\_VENTRICULAR\_CARDIOMYOPATHY\_ARVC |
| Enrichment Score (ES) | -0.37533662 |
| Normalized Enrichment Score (NES) | -1.1836164 |
| Nominal p-value | 0.2514507 |
| FDR q-value | 1.0 |
| FWER p-Value | 0.988 |
Table: GSEA Results Summary

  

Fig 1: Enrichment plot: KEGG\_ARRHYTHMOGENIC\_RIGHT\_VENTRICULAR\_CARDIOMYOPATHY\_ARVC      
 Profile of the Running ES Score & Positions of GeneSet Members on the Rank Ordered List

  

| SYMBOL | TITLE | RANK IN GENE LIST | RANK METRIC SCORE | RUNNING ES | CORE ENRICHMENT || 1 | ITGA4 | na | 272 | 0.550 | 0.0542 | No |
| 2 | CACNB1 | na | 1069 | 0.381 | 0.0742 | No |
| 3 | CACNG8 | na | 1424 | 0.344 | 0.1028 | No |
| 4 | CACNA1F | na | 1554 | 0.333 | 0.1365 | No |
| 5 | CACNA2D4 | na | 6234 | 0.131 | 0.0163 | No |
| 6 | ITGB6 | na | 7342 | 0.105 | -0.0039 | No |
| 7 | CACNG5 | na | 8337 | 0.086 | -0.0229 | No |
| 8 | CACNA2D3 | na | 8962 | 0.075 | -0.0324 | No |
| 9 | ITGA10 | na | 9425 | 0.068 | -0.0380 | No |
| 10 | ATP2A2 | na | 9649 | 0.065 | -0.0371 | No |
| 11 | DSC2 | na | 10122 | 0.059 | -0.0441 | No |
| 12 | ITGB7 | na | 10543 | 0.053 | -0.0502 | No |
| 13 | TCF7L2 | na | 10815 | 0.050 | -0.0524 | No |
| 14 | DAG1 | na | 11829 | 0.038 | -0.0774 | No |
| 15 | ITGB4 | na | 11987 | 0.036 | -0.0778 | No |
| 16 | DSP | na | 12631 | 0.030 | -0.0930 | No |
| 17 | ACTB | na | 12961 | 0.027 | -0.0994 | No |
| 18 | ACTN4 | na | 13347 | 0.024 | -0.1078 | No |
| 19 | ACTN1 | na | 13743 | 0.021 | -0.1168 | No |
| 20 | CTNNB1 | na | 14653 | 0.014 | -0.1415 | No |
| 21 | LMNA | na | 14922 | 0.012 | -0.1479 | No |
| 22 | CTNNA1 | na | 15057 | 0.011 | -0.1505 | No |
| 23 | EMD | na | 15650 | 0.006 | -0.1669 | No |
| 24 | DES | na | 17908 | -0.010 | -0.2310 | No |
| 25 | TCF7 | na | 18185 | -0.012 | -0.2376 | No |
| 26 | ITGA9 | na | 18231 | -0.012 | -0.2376 | No |
| 27 | CACNG3 | na | 18568 | -0.014 | -0.2457 | No |
| 28 | GJA1 | na | 18622 | -0.014 | -0.2456 | No |
| 29 | CDH2 | na | 19001 | -0.017 | -0.2546 | No |
| 30 | JUP | na | 19046 | -0.017 | -0.2539 | No |
| 31 | ITGA2 | na | 19176 | -0.018 | -0.2556 | No |
| 32 | ITGA3 | na | 19278 | -0.019 | -0.2564 | No |
| 33 | CACNB4 | na | 19492 | -0.020 | -0.2602 | No |
| 34 | CACNA1D | na | 19640 | -0.021 | -0.2621 | No |
| 35 | ACTN2 | na | 19694 | -0.022 | -0.2612 | No |
| 36 | ITGA2B | na | 19828 | -0.023 | -0.2624 | No |
| 37 | ACTG1 | na | 20219 | -0.025 | -0.2708 | No |
| 38 | ITGA6 | na | 22097 | -0.041 | -0.3204 | No |
| 39 | CACNB2 | na | 22149 | -0.041 | -0.3172 | No |
| 40 | CACNG1 | na | 22207 | -0.042 | -0.3141 | No |
| 41 | DMD | na | 22362 | -0.044 | -0.3137 | No |
| 42 | ITGAV | na | 23305 | -0.053 | -0.3348 | No |
| 43 | CACNG2 | na | 23379 | -0.054 | -0.3308 | No |
| 44 | SGCD | na | 23777 | -0.060 | -0.3355 | No |
| 45 | TCF7L1 | na | 24530 | -0.070 | -0.3494 | No |
| 46 | SGCB | na | 25142 | -0.078 | -0.3582 | No |
| 47 | CACNA1C | na | 25293 | -0.080 | -0.3535 | No |
| 48 | ITGA7 | na | 26050 | -0.091 | -0.3651 | Yes |
| 49 | CACNA2D2 | na | 26358 | -0.096 | -0.3631 | Yes |
| 50 | ITGA11 | na | 26468 | -0.098 | -0.3552 | Yes |
| 51 | CTNNA2 | na | 26542 | -0.099 | -0.3461 | Yes |
| 52 | CACNB3 | na | 26714 | -0.103 | -0.3395 | Yes |
| 53 | ITGB5 | na | 27213 | -0.113 | -0.3412 | Yes |
| 54 | CACNG7 | na | 27851 | -0.127 | -0.3452 | Yes |
| 55 | ITGB1 | na | 28114 | -0.134 | -0.3377 | Yes |
| 56 | PKP2 | na | 28865 | -0.154 | -0.3420 | Yes |
| 57 | SLC8A1 | na | 29232 | -0.164 | -0.3341 | Yes |
| 58 | ITGB3 | na | 29449 | -0.171 | -0.3211 | Yes |
| 59 | ITGB8 | na | 29484 | -0.172 | -0.3027 | Yes |
| 60 | CACNG4 | na | 29566 | -0.174 | -0.2854 | Yes |
| 61 | DSG2 | na | 29603 | -0.175 | -0.2667 | Yes |
| 62 | CACNA2D1 | na | 29712 | -0.179 | -0.2496 | Yes |
| 63 | ITGA8 | na | 29843 | -0.183 | -0.2328 | Yes |
| 64 | ITGA1 | na | 29976 | -0.187 | -0.2155 | Yes |
| 65 | SGCG | na | 31057 | -0.222 | -0.2216 | Yes |
| 66 | LAMA2 | na | 31294 | -0.231 | -0.2024 | Yes |
| 67 | CACNG6 | na | 31816 | -0.253 | -0.1889 | Yes |
| 68 | SGCA | na | 32440 | -0.283 | -0.1750 | Yes |
| 69 | ITGA5 | na | 32559 | -0.291 | -0.1455 | Yes |
| 70 | LEF1 | na | 32575 | -0.292 | -0.1130 | Yes |
| 71 | CTNNA3 | na | 32684 | -0.300 | -0.0823 | Yes |
| 72 | RYR2 | na | 33835 | -0.395 | -0.0710 | Yes |
| 73 | CACNA1S | na | 33887 | -0.403 | -0.0271 | Yes |
| 74 | ACTN3 | na | 34217 | -0.455 | 0.0147 | Yes |
Table: GSEA details [plain text format]

  

Fig 2: KEGG\_ARRHYTHMOGENIC\_RIGHT\_VENTRICULAR\_CARDIOMYOPATHY\_ARVC      
 Blue-Pink O' Gram in the Space of the Analyzed GeneSet

  

Fig 3: KEGG\_ARRHYTHMOGENIC\_RIGHT\_VENTRICULAR\_CARDIOMYOPATHY\_ARVC: Random ES distribution      
 Gene set null distribution of ES for **KEGG\_ARRHYTHMOGENIC\_RIGHT\_VENTRICULAR\_CARDIOMYOPATHY\_ARVC**
